# [^18^F]Fluoronicotinic-Acid-Conjugated Folate as a Novel Candidate Positron Emission Tomography Tracer for Inflammation

**DOI:** 10.1021/acsomega.5c10157

**Published:** 2025-12-31

**Authors:** Xiaoqing Zhuang, Jonne Kunnas, David Ekwe, Emel Bakay, Pyry Dillemuth, Heidi Liljenbäck, Imran Iqbal, Johan Rajander, Philip S. Low, Juhani Knuuti, Jessica M. Rosenholm, Antti Saraste, Anne Roivainen, Xiang-Guo Li

**Affiliations:** ‡ Turku PET Centre, 8058 University of Turku, Kiinamyllynkatu 4-8, FI-20520 Turku, Finland; § Turku PET Centre, Turku University Hospital, Kiinamyllynkatu 4-8, FI-20520 Turku, Finland; ∥ Department of Chemistry, University of Turku, Henrikinkatu 2, FI-20500 Turku, Finland; ⊥ Pharmaceutical Sciences Laboratory, Department of Natural and Health Sciences, Faculty of Science and Engineering, 1040 Åbo Akademi University, Tykistökatu 6A, FI-20521 Turku, Finland; # Accelerator Laboratory, Åbo Akademi University, Kiinamyllynkatu 4-8, FI-20520 Turku, Finland; ∇ Department of Chemistry, 311308 Purdue University, 560 Oval Drive, West Lafayatte, Indiana 47907-2084, United States; ○ InFLAMES Research Flagship Center, University of Turku, Tykistökatu 6A, FI-20521 Turku, Finland; ◆ Heart Center, Turku University Hospital, Hämeentie 11, FI-20520 Turku, Finland; ¶ Turku Center for Disease Modeling, University of Turku, Kiinamyllynkatu 10, FI-20520 Turku, Finland

## Abstract

Folate receptors are clinically relevant targets, as evidenced by therapeutic agents, including mirvetuximab soravtansine-gynx, an antibody–drug conjugate recently approved for cancer treatment. In this study, we report the development of a novel positron emission tomography (PET) imaging agent, [^18^F]­fluoronicotinic-acid-conjugated folate ([^18^F]­FNA–folate), for the evaluation of folate receptor expression. The [^18^F]­FNA–folate was synthesized via the N-acylation of an amino-functionalized folate derivative with [^18^F]­FNA 4-nitrophenyl ester under mild reaction conditions. The resulting radiotracer exhibited excellent *in vitro* and *in vivo* stability, rapid blood clearance, and minimal bone uptake in mice and rats. *In vitro* tissue binding studies using heart sections from an experimental rat model of myocardial infarction demonstrated focal, intense, and heterogeneous uptake of [^18^F]­FNA–folate, and the binding specificity to macrophage folate receptors was confirmed. The straightforward radiosynthesis, excellent *in vivo* stability, and target-specific binding support further development of [^18^F]­FNA–folate as a promising PET imaging agent for inflammatory diseases.

## Graphical abstract

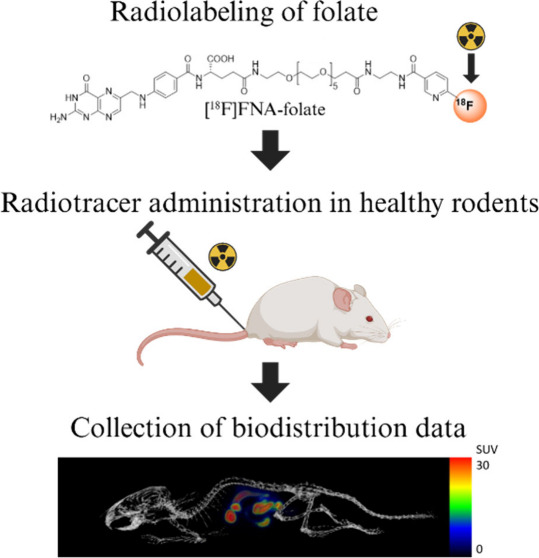

## Introduction

Theranostics for precision medicine is increasingly being translated into clinical practice. Folate receptors are upregulated in several diseases, providing opportunities for developing new theranostic agents. In the recent Theranostic Genome study,^1^ folate receptors were confirmed as potential targets for theranostics. This finding aligns with promising preclinical and clinical data on folate receptor-targeted imaging and radiotherapy.^2^ Significant efforts in the international research community have been devoted to the development of new drugs targeting folate receptors, leading to important breakthroughs. In 2022, mirvetuximab soravtansine-gynx, a folate receptor-α–mediated antibody–drug conjugate, was approved by the United States Food and Drug Administration for the treatment of platinum-resistant epithelial ovarian and other cancers.^3^

Several agents have shown encouraging preclinical and clinical results in the area of folate receptor-targeted radiopharmaceuticals.^4–6^ We have focused on the development of fluorine-18 (^18^F)- and gallium-68-labeled folate tracers for positron emission tomography (PET) imaging of inflammation.^6–8^ The radiolabeling of biomolecules with ^18^F typically requires a prosthetic group, which should be straightforward to synthesize, exhibit high *in vivo* stability, and enable robust and scalable manufacturing. [^18^F]­Fluoronicotinic acid ([^18^F]­FNA) has emerged as a favorable prosthetic group for radiolabeling peptides and antibody fragments,^9–11^ with clinical relevance demonstrated by the success of [^18^F]­FNA conjugates such as Pylarify (piflufolastat F-18) and [^18^F]-PSMA-1007 for prostate cancer imaging.^12,13^

Activated esters of [^18^F]­FNA, such as [^18^F]­FNA 4-nitrophenyl ester, enable efficient N-acylation of amino-functionalized biomolecules. Compared with earlier prosthetic agents, such as *N*-succinimidyl-4-[^18^F]­fluorobenzoate ([^18^F]­SFB), the radiosynthesis of [^18^F]­FNA esters is considerably more straightforward. We have been focusing on the use of [^18^F]­FNA 4-nitrophenyl ester as a prosthetic group for radiolabeling biomolecules for PET applications.^10,11,14^ Notably, the [^18^F]­FNA 4-nitrophenyl ester reacts with amino groups to form N-acylated conjugates and can also undergo highly selective S-acylation with thiol groups.^11^ In addition, the [^18^F]­FNA 4-nitrophenyl ester is conveniently produced using conventional K­[^18^F]­F/K_2.2.2_-based nucleophilic substitution or on-resin ^18^F-fluorination, and its nonvolatility improves radiation safety.

In this study, we report the preparation of [^18^F]­FNA–folate using [^18^F]­FNA 4-nitrophenyl ester as the ^18^F-prosthetic group for conjugation with an amino-functionalized folate derivative (Figure 1). Furthermore, we evaluated the *in vivo* stability and biodistribution of [^18^F]­FNA–folate in healthy rats and mice, as well as its binding in heart tissue sections from a rat model of myocardial infarction-induced inflammation.

**Figure 1:**
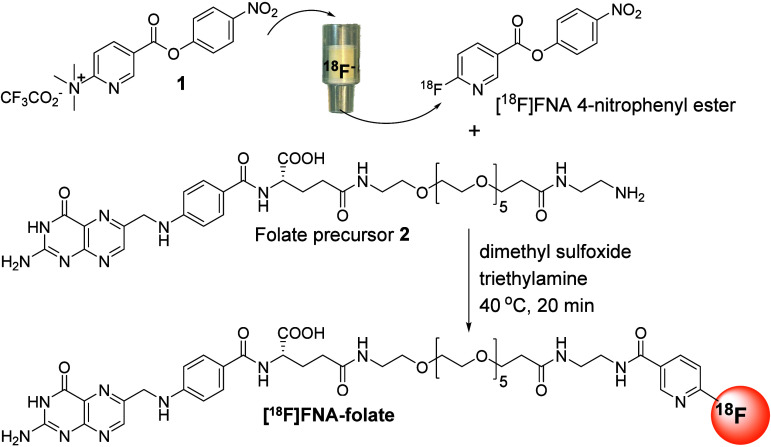
Chemical scheme for the preparation of [^18^F]­FNA–folate. The prosthetic compound [^18^F]­FNA 4-nitrophenyl ester was prepared by on-resin ^18^F-fluorination of compound **1** and conjugation with folate precursor **2** in the presence of triethylamine as a base.

## Results and Discussion

### Radiosynthesis and Characterization of [^18^F]­FNA–Folate

To prepare [^18^F]­FNA–folate, the first step involved synthesizing the prosthetic compound [^18^F]­FNA 4-nitrophenyl ester (Figure 1). Similar to previous reports,^11^ [^18^F]­FNA 4-nitrophenyl ester was prepared by passing a solution of precursor compound **1** through an anion-exchange cartridge (PS-HCO_3_^–^), which binds [^18^F]­fluoride to the sorbent. In this process, nucleophilic ^18^F-fluorination occurs on resin inside the cartridge, eliminating the need for azeotropic drying of [^18^F]­fluoride. This approach is straightforward and can be automated. Typically, 5–10 mg of precursor compound **1** was required per batch to ensure efficient ^18^F-fluorination. The [^18^F]­FNA 4-nitrophenyl ester was isolated using semi-preparative high-performance liquid chromatography (HPLC) on a reversed-phase C18 column and subsequently extracted onto a hydrophilic–lipophilic balance (HLB) solid-phase extraction cartridge. Finally, the [^18^F]­FNA 4-nitrophenyl ester was eluted into a reaction vial with a small volume (350 μL) of acetonitrile. In general, to facilitate the efficient conjugation of prosthetic compounds with biomolecules, it is essential that the prosthetic compounds are concentrated in small volumes to reduce the amount of input biomolecules in the reaction.^15^ In this case, the use of 350 μL acetonitrile to formulate the purified prosthetic group [^18^F]­FNA 4-nitrophenyl ester was appropriate because the amount of acetonitrile was sufficient to elute >90% of the radioactivity out of the HLB cartridge and the protocol is highly reproducible. Thus far, using this radiosynthesis procedure, we have robustly produced [^18^F]­FNA 4-nitrophenyl ester for different purposes, including the synthesis of [^18^F]­FNA–folate, as described below.

The next step involved conjugating the [^18^F]­FNA 4-nitrophenyl ester with the amino-functionalized folate precursor compound **2** via an N-acylation reaction (Figure 1). The initial tests were performed in borate buffer at pH 8.6 under reaction conditions similar to those previously reported for peptide conjugation.^10^ However, only a negligible amount of [^18^F]­FNA–folate was formed, and the reaction remained sluggish even at an elevated temperature (50 °C). This outcome contrasted with our previous peptide conjugation, in which the reaction was completed within 5 min at room temperature (RT).^10^ In the peptide sequence, the first amino acid residue at the N-terminus is cysteine, which bears a free sulfhydryl group in close proximity to the free amino group. The N-acylation of the peptide with [^18^F]­FNA 4-nitrophenyl ester proceeded surprisingly fast at RT and with nearly exclusive chemoselectivity. We hypothesized that peptide N-acylation was facilitated by an intramolecular *S*- to *N*-acyl transfer. In folate precursor **2**, no such thiol group was available to mediate the acyl transfer reaction, which might be a factor underlying the observed sluggish N-acylation in the radiosynthesis of [^18^F]­FNA–folate.

Therefore, we tested the use of the organic base triethylamine in dimethyl sulfoxide (DMSO) as the reaction medium, which has previously been used in N-acylation of biomolecules and small organic compounds.^16,17^ This significantly improved the conjugation efficiency. [^18^F]­FNA–folate was obtained with a decay-corrected radiochemical yield of 16.8 ± 10.0% (*n* = 6) and high radiochemical purity (97.2 ± 1.2%). The molar activity was 68.0 ± 24.9 GBq/μmol (*n* = 4) at the end of synthesis. To prevent radiolysis, it was essential to include ascorbic acid (16 mM) in the final formulation. In our experience, radiolysis has been observed in all folate-derived radiotracers.^6,8^ Folate is a vitamin that tends to be oxidized, and ascorbic acid is an effective antioxidant that protects folate from oxidation.^18^ We found that ascorbic acid is also effective in the prevention of [^18^F]­FNA–folate radiolysis. The pH of the end product was adjusted to 6–7 in phosphate-buffered saline (PBS) containing less than 10% ethanol (EtOH), making it suitable for *in vivo* PET imaging applications. The total synthesis time was 170 ± 10 min (*n* = 6), starting from the end of bombardment. The [^18^F]­FNA–folate remained stable in the end product formulation for at least 6 h (longer times were not tested), as confirmed by HPLC analysis (Figure 2).

**Figure 2:**
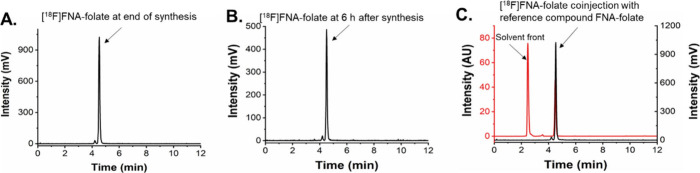
HPLC analysis of the radiochemical purity and chemical identity of [^18^F]­FNA–folate. Radiochemical purity (A) at the end of synthesis and (B) 6 h after synthesis. (C) Chemical identity of [^18^F]­FNA–folate was confirmed by HPLC comparison with FNA–folate as the standard under the same analytical conditions. In panel C, the HPLC trace of FNA–folate was shown in red and [^18^F]­FNA–folate was shown in black.

To confirm the chemical identity of [^18^F]­FNA–folate, the non-radioactive reference compound FNA–folate was prepared via N-acylation using FNA 4-nitrophenyl ester as the acyl donor and folate precursor **2** as the acyl acceptor under mild conditions (40 °C) similarly as in the radiosynthesis. The product was isolated by semi-preparative HPLC, and its identity was confirmed by electrospray ionization mass spectrometry (ESI–MS) analyses. The calculated mass of the parent molecule C_42_H_56_FN_11_O_13_ was 941.97, and the observed mass (942.90 [M + H]^+^) closely matched the expected value. HPLC analysis of [^18^F]­FNA–folate spiked with the reference FNA–folate showed that both compounds eluted at the same retention time (Figure 2C), confirming the radiotracer’s chemical identity. The distribution coefficient log *D*_7.4_ of [^18^F]­FNA–folate was −3.39 ± 0.04 (*n* = 3), indicating that the compound was highly hydrophilic.

### PET Imaging and Biodistribution in Healthy Rats and Mice

After the successful radiosynthesis of [^18^F]­FNA–folate, its biological properties were evaluated in both *in vivo* and *in vitro* preclinical settings. The study design was shown in Figure S1. PET imaging was performed in healthy Sprague–Dawley rats (*n* = 4, male, age 7.1 ± 0.0 weeks, bodyweight 232.6 ± 14.4 g) following intravenous injection of [^18^F]­FNA–folate at a dose of 10.27 ± 0.15 MBq per rat. Dynamic PET images were acquired for 60 min, and computed tomography (CT) was used for anatomical reference and attenuation correction. PET images were analyzed using our in-house software Carimas 2.10. In rats, [^18^F]­FNA–folate exhibited rapid kinetics and was primarily excreted via the liver and kidneys (Figure 3). The uptake in the renal cortex of both kidneys was clearly visible (Figure 3). Time–activity curves (TACs, Figure 4) showed rapid clearance from the blood circulation, and low uptake in most organs, except for high accumulation and retention in the kidneys. During the last 30 min of imaging, the mean standardized uptake values (SUV_mean_) in the kidneys, liver, heart (whole heart), muscle, lungs, and brain were 11.44 ± 0.94, 1.01 ± 0.06, 0.28 ± 0.02, 0.28 ± 0.03, 0.22 ± 0.02, and 0.10 ± 0.01, respectively. Bone uptake was relatively low, with SUV_mean_ in trabecular and cortical bones of 0.59 ± 0.08 and 0.43 ± 0.04, respectively. Bone TACs showed declining trends over time, indicating clearance rather than accumulation and that *in vivo* ^18^F-defluorination was not significant (if any).

**Figure 3:**
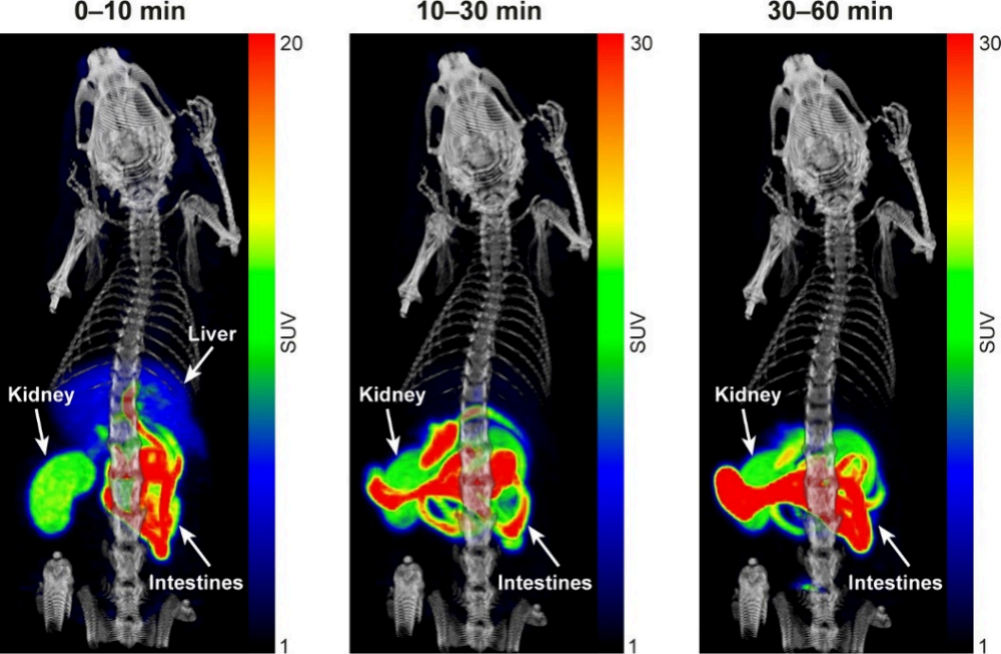
Coronal PET/CT images (maximum intensity projection) of a rat injected with [^18^F]­FNA–folate, showing weighted mean standardized uptake values (SUV_mean_) at 1–10, 10 – 30, and 30–60 min post-injection, respectively.

**Figure 4:**
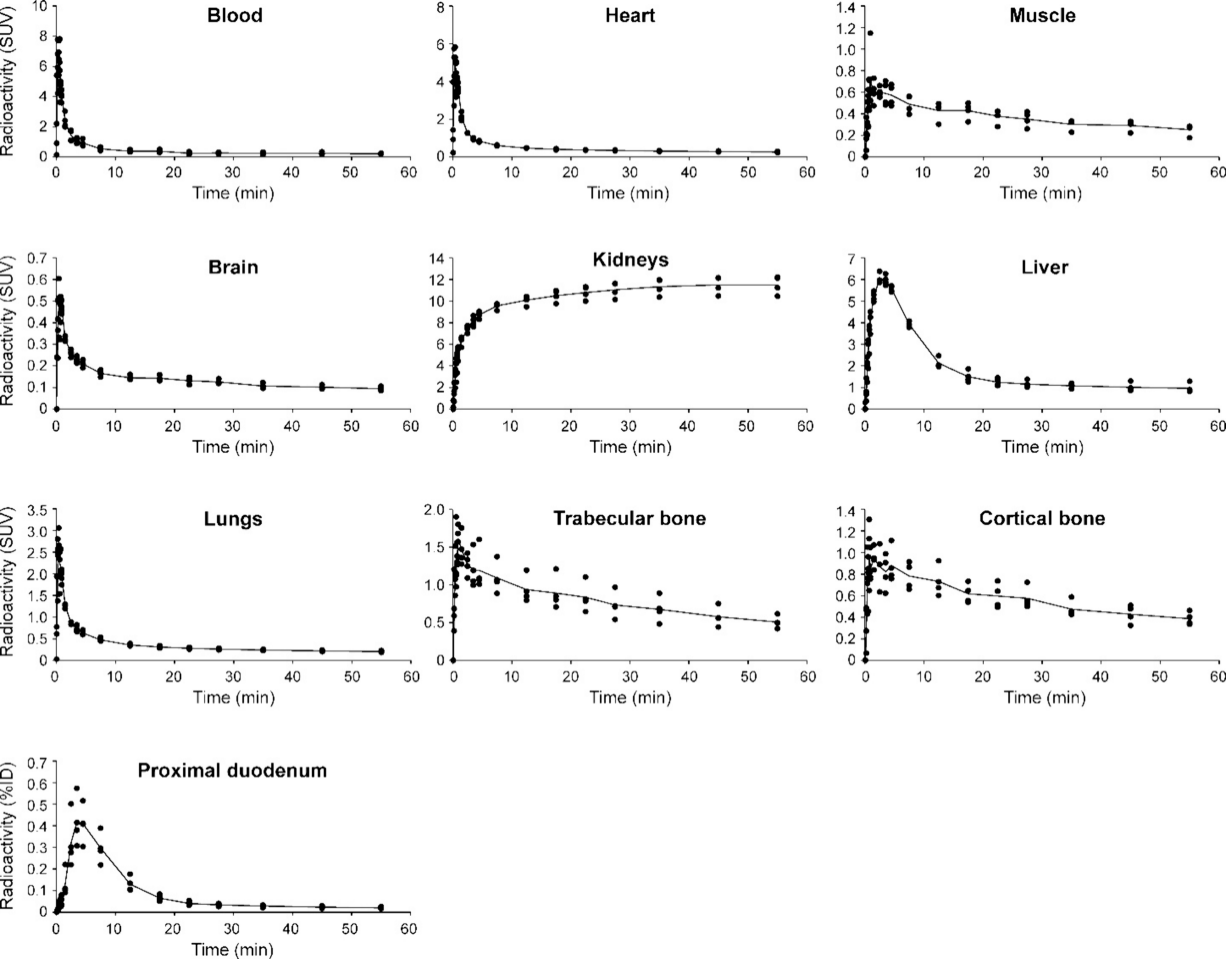
Time–activity curves (TACs) of selected organs and tissues in healthy rats injected with [^18^F]­FNA–folate (*n* = 4). Heart radioactivity was quantified from the whole heart volume.

Similar PET/CT imaging experiments were performed in healthy C57BL/6J mice (*n* = 8, males, age 13.1 ± 1.0 weeks, bodyweight 31.8 ± 3.1 g) following intravenous injection of [^18^F]­FNA–folate at doses of 4.06 ± 0.42 MBq. The tracer uptake kinetics were comparable to those observed in rats. Radioactivity was rapidly cleared from the blood pool, and the main excretion route was the urinary tract (Figures 5 and 6). Immediately after the 60 min dynamic PET imaging, four of the mice were sacrificed for tissue collection. Tissue-associated radioactivity was measured by gamma counting and expressed as the percentage of injected radioactivity dose per gram (%ID/g) of tissue (Figure 7). The radioactivity concentration in the kidneys, liver, and urine was 20.31 ± 2.28 (*n* = 4), 2.90 ± 2.64 (*n* = 4), and 11.13 ± 4.99 (*n* = 4), respectively. Among all samples, the lowest values were observed in blood (0.03 ± 0.01; *n* = 4), heart (0.02 ± 0.01; *n* = 4), and brain (0.04 ± 0.01; *n* = 4). The uptake in skull bone and femur (bone + bone marrow) was also low at 0.25 ± 0.21 (*n* = 4) and 0.90 ± 1.51 (*n* = 4), respectively. These findings provide further evidence that the *in vivo* ^18^F-defluorination of the tracer was not significant. In addition to the *ex vivo* biodistribution measurements described above, *in vivo* biodistribution was also quantified based on the 60 min dynamic PET imaging and was presented as %ID/mL (Table S1).

**Figure 5:**
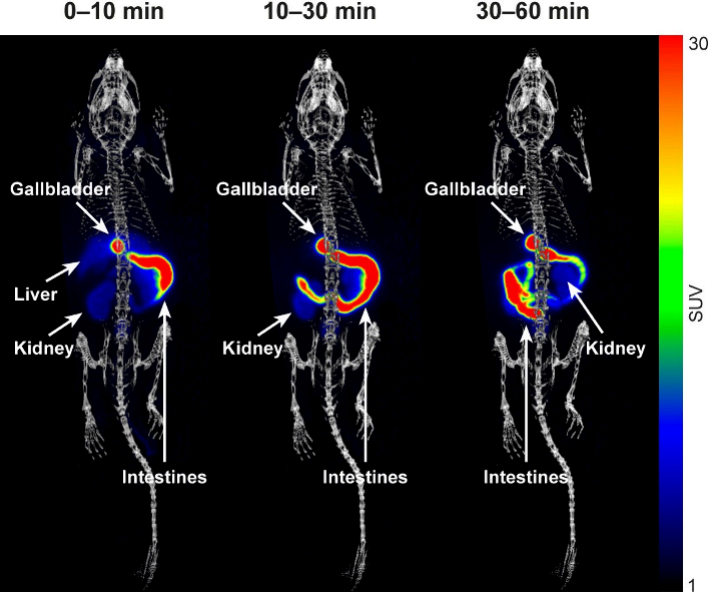
Coronal PET/CT images (maximum intensity projection) of a mouse injected with [^18^F]­FNA–folate, showing weighted mean standardized uptake values (SUV_mean_) at 1–10, 10 – 30, and 30–60 min post-injection, respectively.

**Figure 6:**
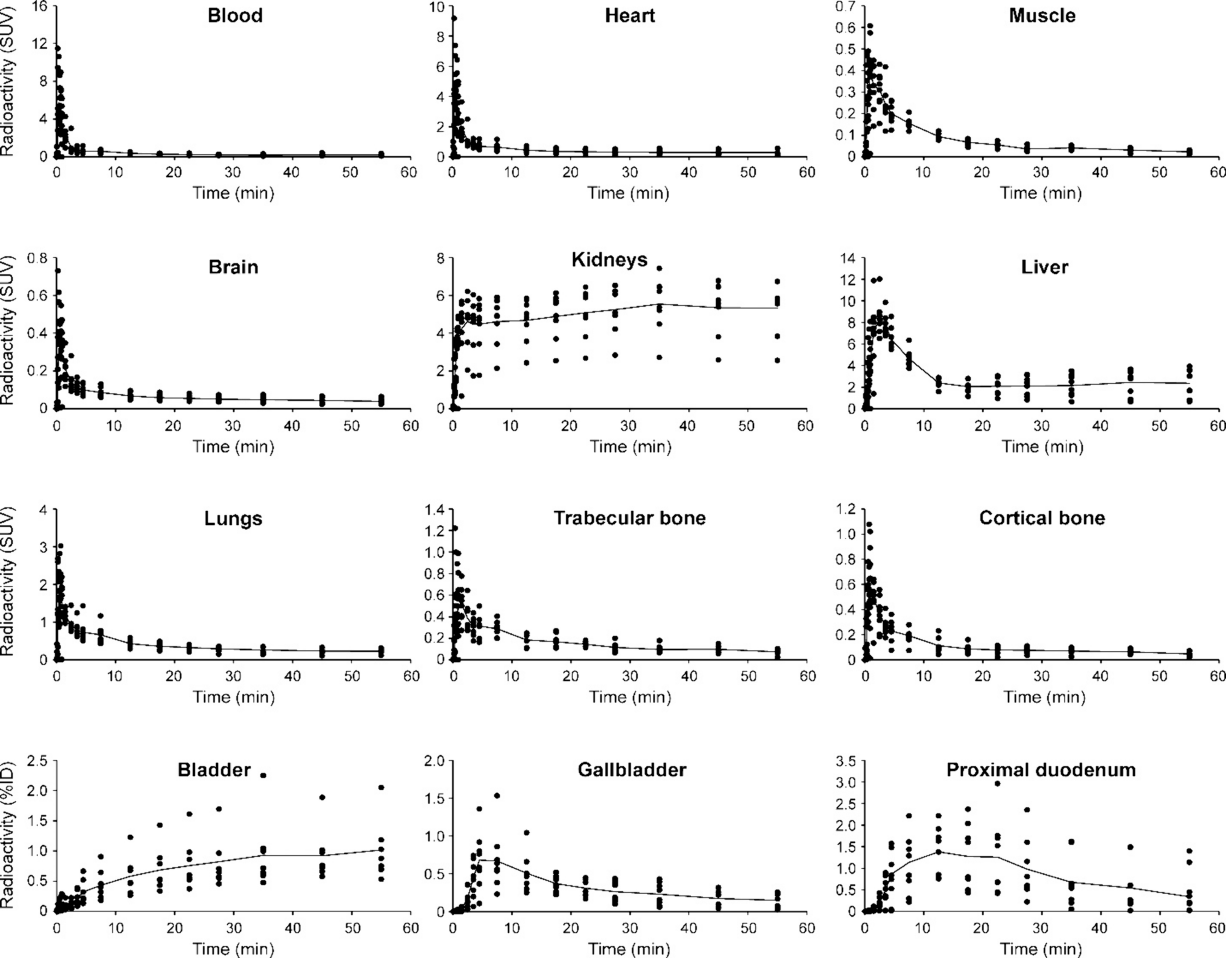
Time–activity curves (TACs) of selected organs and tissues in healthy mice injected with [^18^F]­FNA–folate (*n* = 8). Heart radioactivity was quantified from the entire heart volume.

**Figure 7:**
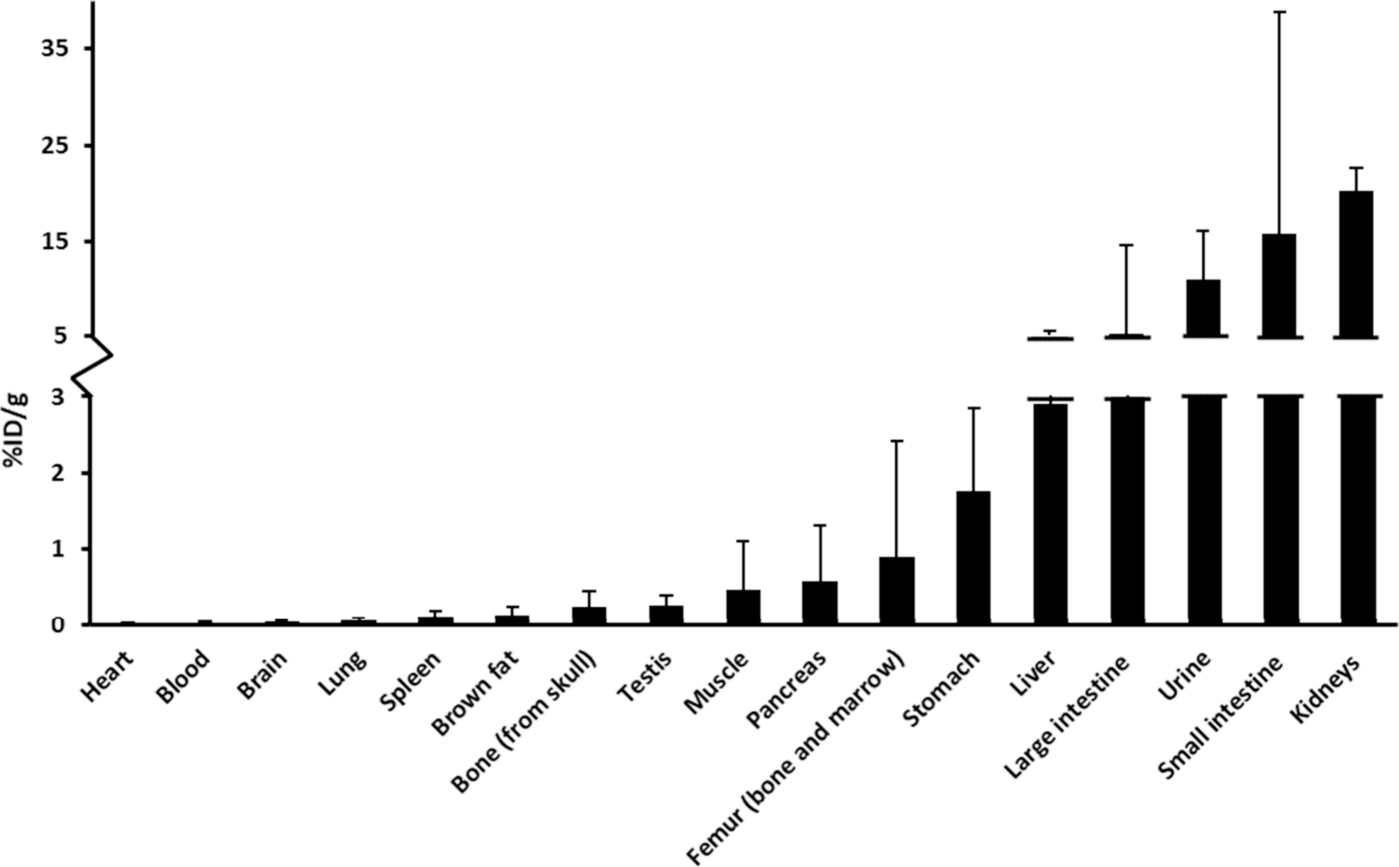
*Ex vivo* tissue biodistribution of [^18^F]­FNA–folate in mice at 60 min post-injection (*n* = 4). Stomach and intestines were measured after removal of contents.

### *In Vivo* Stability and Radioactivity Analysis of Blood

In the development of new radiopharmaceuticals, *in vivo* stability is a critical parameter to evaluate. Accordingly, blood samples were collected 60 min after the injection of [^18^F]­FNA–folate in rats. Blood cells, plasma proteins, and plasma supernatant were separated to quantify radioactivity. In rats, the distribution of blood radioactivity was 12.8 ± 1.7% (*n* = 4) in blood cells, 38.2 ± 6.2% (*n* = 4) bound to plasma proteins, and 49.0 ± 6.2% (*n* = 4) in plasma supernatant. In mice, the corresponding values were 24.4 ± 7.3% (*n* = 4), 9.6 ± 6.5% (*n* = 4), and 66.0 ± 6.5% (*n* = 4), respectively. Plasma supernatant samples were further analyzed by HPLC, and intact [^18^F]­FNA–folate was identified by comparison with the tracer standard (Figure 8). At 60 min post-injection, 95.0 ± 3.9% (*n* = 4) of the tracer remained intact, indicating excellent *in vivo* stability. In mice, [^18^F]­FNA–folate also demonstrated high stability in tests similar to those performed in rats. We did not find it necessary to evaluate the *in vivo* stability at longer time points than 60 min, as this time frame is generally sufficient for clinical diagnostic PET imaging in most cases. The prosthetic group [^18^F]­FNA is a fluorinated analogue of niacin, one of the three forms of vitamin B_3_. We have recently shown that [^18^F]­FNA can be used for PET imaging of intracranial glioblastoma xenografts.^14,19^ In cases where [^18^F]­FNA appears as a major radiometabolite of [^18^F]­FNA-conjugated biomolecules, its cofounding effects on target imaging and tissue uptake kinetics must be considered.^14^ However, in the case of [^18^F]­FNA–folate, the excellent *in vivo* stability simplifies both radioactivity quantification and data interpretation. PET quantification is based on radioactivity detection, thus being a summation of all the radioactive chemical entities, including the intact tracer and its radioactive metabolites. In tracer kinetic analysis, the radioactivity concentration in tissues should be corrected using the percentage of intact tracer relative to total radioactivity. There is no commonly defined tracer stability threshold for radioactivity correction purposes, the reason for which is that it is dependent on the study’s purpose in individual projects. In some cases, performing radioactivity correction is not considered necessary if the amount of radiometabolite is <10%.^20^ In this study, we did not find radioactivity correction necessary in the tissue uptake kinetic analysis. Furthermore, this work demonstrates that the instability of [^18^F]­FNA-conjugated biomolecules is not an inherent property of [^18^F]­FNA as a prosthetic group but rather depends on the stability of the biomolecule itself. It is worth keeping this point in mind when designing radiolabeling for new biomolecules with [^18^F]­FNA as a prosthetic group.

**Figure 8:**
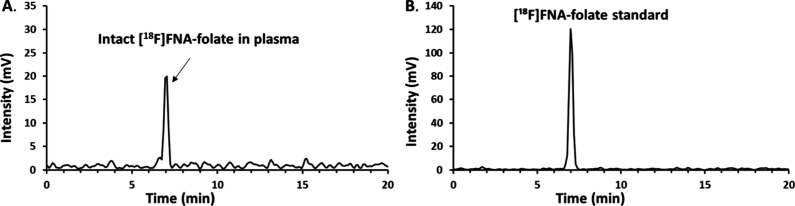
*In vivo* stability of [^18^F]­FNA–folate analyzed by HPLC. (A) Mouse plasma sample collected 60 min post-injection. (B) [^18^F]­FNA–folate tracer standard.

### *In Vitro* Binding in Heart Tissue Sections from Rats with Myocardial Infarction

To evaluate the potential of [^18^F]­FNA–folate for inflammation imaging, *in vitro* binding studies were performed using rat heart cryosections obtained from a myocardial infarction model.^21^ The rat model was prepared by a surgical procedure, and tissue inflammation involving macrophage activation was observed in the healing process after the experimental myocardial infarction. The heart sections were incubated with [^18^F]­FNA–folate in PBS for 45 min, following a previously reported protocol for *in vitro* tissue binding.^10^ The sections were then washed, dried, and subjected to autoradiographic imaging alongside histological and immunohistochemical staining (Figure 9). Histological analysis revealed inflammatory lesions that were readily detectable by hematoxylin and eosin (H&E) staining (Figure 9A). These lesions co-localized with CD68-positive macrophages (Figure 9B) and exhibited focal and intense radioactivity binding (Figure 9C). CD68, a pan-macrophage marker expressed in both M1 and M2 macrophages as well as other immune cells, confirmed macrophage involvement in the lesions.

**Figure 9:**
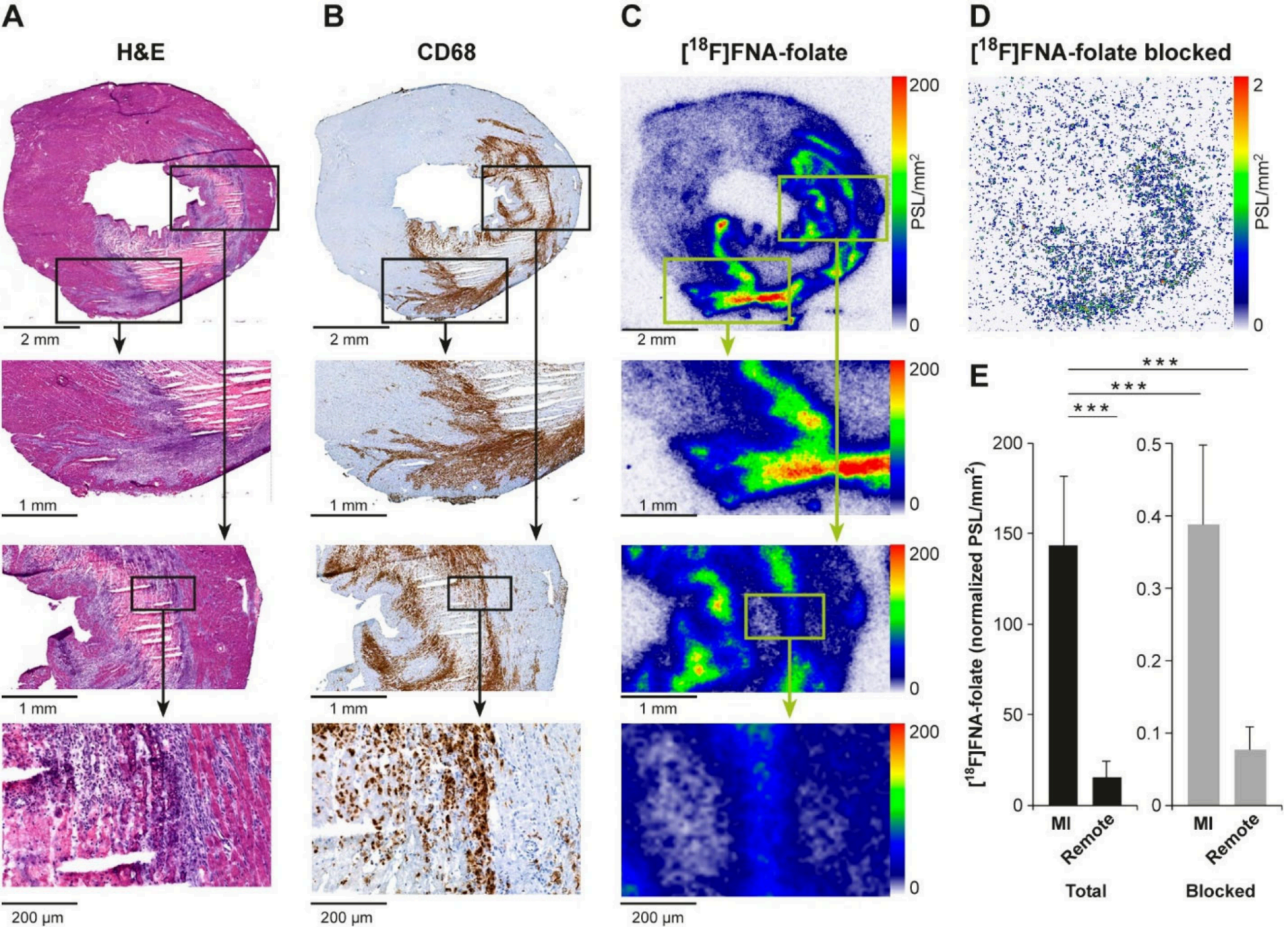
Histological, immunohistochemical, and autoradiographic analysis of rat heart tissue cryosections. (A) H&E staining revealed myocardial infarction-induced inflammatory lesions. (B) Immunohistochemistry showed activated macrophages within the infarcted (inflamed) regions. (C) Autoradiography demonstrated focal and intense binding of [^18^F]­FNA–folate in adjacent tissue sections, consistent with macrophage-rich inflammatory sites. (D) *In vitro* blocking experiments were performed on adjacent tissue sections in the presence of folate glucosamine. (E) Intensity of [^18^F]­FNA–folate *in vitro* total and blocked binding in myocardial infarction (MI)-induced inflamed tissue was significantly higher than in remote tissues.

To confirm receptor-mediated binding, competitive blocking studies were performed on adjacent tissue sections using a mixture of [^18^F]­FNA–folate and folate glucosamine (1 μM) as a blocking agent. In our previous studies, folate glucosamine effectively inhibited folate receptor-β binding on activated macrophages during inflammation.^6,7^ Consistently, folate glucosamine reduced [^18^F]­FNA–folate binding by >99% (*n* = 17), with residual tissue binding approaching background levels (Figure 9D). In the *in vitro* blocking experiments using rat heart cryosections from a myocardial infarction model, the intensity of radioactivity binding in infarct and remote regions were 0.39 ± 0.11 PSL/mm^2^ (*n* = 17) and 0.08 ± 0.03 PSL/mm^2^, respectively. In comparison, in the total binding experiments, the corresponding values were 143.68 ± 37.70 PSL/mm^2^ (*n* = 17) and 15.53 ± 8.88 PSL/mm^2^, respectively (Figure 9E).

### Study Limitations

The current radiosynthesis of [^18^F]­FNA–folate requires approximately 3 h and involves two HPLC purification steps. While this procedure is suitable for preclinical studies, it may present challenges for fully automated manufacturing under good manufacturing practice (GMP) conditions. Nevertheless, given the successful GMP production of the [^18^F]­SFB-conjugated folate tracer,^22^ it is reasonable to anticipate that the [^18^F]­FNA–folate synthesis could be simplified by omitting at least one HPLC purification step, although this has not been the focus of the current study. Importantly, we demonstrated that [^18^F]­FNA–folate tissue binding was reduced by >99% in the presence of folate glucosamine, providing strong evidence that the use of [^18^F]­FNA as a prosthetic group preserves folate receptor binding properties. However, the current experiments do not clarify whether [^18^F]­FNA–folate exhibits subtype selectivity toward different folate receptor isoforms.

## Conclusion

A novel PET imaging agent, [^18^F]­FNA–folate, was successfully synthesized using [^18^F]­FNA as the prosthetic group. The tracer demonstrated excellent *in vitro* and *in vivo* stability and rapid clearance from the blood circulation. In myocardial infarction rat tissue samples, [^18^F]­FNA–folate exhibited intense and specific binding to macrophage-associated folate receptors. Thus, it is feasible to use [^18^F]­FNA as a prosthetic group to radiolabel folate for targeted PET imaging. The application of [^18^F]­FNA–folate in disease models with well-characterized folate receptor expression, such as cancer, atherosclerosis, and arthritis, may further establish its potential as a clinically relevant imaging agent.

## Methods

### Materials

*N*,*N*,*N*-Trimethyl-5-((4-nitrophenoxy)­carbonyl)­pyridine-2-aminium triflate (**1**) was purchased from R&S Chemicals (Kannapolis, NC, U.S.A.). The folate precursor compound **2** was purchased from ABX GmbH (Radeberg, Germany). The nonradioactive reference compound FNA 4-nitrophenyl ester was purchased from Enamine (Kyiv, Ukraine). All other chemicals and solvents were purchased from Merck (Darmstadt, Germany).

### Synthesis of the Reference FNA–Folate

Triethylamine (1.38 μL) was added to a solution of FNA 4-nitrophenyl ester (48 μg, 0.18 μmol) and folate precursor **2** (0.15 mg, 0.18 μmol) in 150 μL of DMSO. The mixture was shaken at 40 °C for 1 h. The product was purified by HPLC on a Jupiter Proteo C18 semi-preparative column (10 μm, 90 Å, 250 × 10 mm, Phenomenex, Torrance, CA, U.S.A.). Solvent A was H_2_O with 0.1% trifluoroacetic acid (TFA), and solvent B was acetonitrile (CH_3_CN) with 0.1% TFA. The elution gradient was 45–70% B over 15 min at a flow rate of 4 mL/min.

### Radiosynthesis of the [^18^F]­FNA–Folate

[^18^F]­Fluoride was trapped on a PS–HCO_3_^–^ cartridge (45 mg, Synthra GmbH, Hamburg, Germany) preconditioned with 1 mL EtOH and 1 mL water. The cartridge was washed with 5 mL of CH_3_CN and dried for 3 min under a nitrogen flow. Precursor **1** (8 mg) was dissolved in 1 mL CH_3_CN/*tert*-butanol (1:4, v/v), and the solution was slowly passed through the PS-HCO_3_^–^ cartridge and collected in a vial. The cartridge was washed with 0.5 mL CH_3_CN into the same vial. The [^18^F]­FNA 4-nitrophenyl ester was purified by HPLC. For HPLC purification, a Jupiter Proteo semi-preparative column (4 μm, 90 Å, 250 × 10 mm; Phenomenex) was used. Solvent A was H_2_O with 0.1% TFA, and solvent B was CH_3_CN with 0.1% TFA. The elution gradient was 45–70% B over 15 min at a flow rate of 4 mL/min. The collected fraction was diluted with 35 mL H_2_O and passed through an Oasis 30 mg HLB cartridge (Waters, Milford, NH, U.S.A.) preconditioned with 10 mL EtOH and 10 mL H_2_O. The HLB cartridge was washed with 5 mL H_2_O, and the bound [^18^F]­FNA 4-phenyl ester was eluted out with 350 μL CH_3_CN into a mixture of folate precursor **2** (4 mg) and triethylamine (30 μL) in DMSO (400 μL). The mixture was heated at 40 °C for 20 min. The reaction was then quenched with 0.38 M HCl (800 μL), and the mixture was injected into HPLC for purification. [^18^F]­FNA–folate was purified using a Jupiter Proteo column (4 μm, 90 Å, 250 × 10 mm; Phenomenex) at a flow rate of 4 mL/min with solvent A (0.1% TFA in water) and solvent B (0.1% TFA in CH_3_CN). The elution program was 10–30% B for 0–15 min, followed by 30% B isocratic for 15–25 min. [^18^F]­FNA–folate was eluted at approximately 17 min.

The collected fraction was diluted with 25 mL H_2_O containing 2.4 mM ascorbic acid, and the product was trapped on a light tC18 cartridge (Waters, Milford, NH, U.S.A.). The product was then eluted with 0.5 mL of 50% EtOH into a vial containing 2 mL saline, 20 mM ascorbic acid, and 100 mM phosphate buffer (500 μL).

The end product [^18^F]­FNA–folate was analyzed by HPLC using a Jupiter Proteo analytical column (4 μm, 90 Å, 250 × 4.6 mm; Phenomenex) with a 23–45% B gradient over 12 min at a flow rate of 1 mL/min. Solvent A was H_2_O with 0.1% TFA, and solvent B was CH_3_CN with 0.1% TFA. Detection was performed with both radioactivity and ultraviolet (UV) absorbance at 280 nm. Shelf life testing of [^18^F]­FNA–folate was performed by sampling up to 6 h post-radiosynthesis, with radiochemical purity assessed by analytical HPLC as described above. The pH of the end product was measured using pH indicator strips.

### Log *D*_7.4_ Measurements

To evaluate the hydrophilicity of [^18^F]­FNA–folate, the distribution coefficient log *D*_7.4_ was measured by adding 5 kBq of [^18^F]­FNA–folate to a mixture of 600 μL of 1-octanol and 600 μL of PBS (pH 7.4). The mixture was thoroughly vortexed for 3 min, followed by centrifugation at 12000*g* for 3 min to separate the phases. Subsequently, 400 μL aliquots were taken from each phase, and radioactivity was quantified using a Triathler 3″ gamma counter (Hidex, Turku, Finland). The experiments were performed in triplicate. Log *D*_7.4_ was calculated using the following formula: $\log D=\log_{10}\frac{\mathrm{counts in octanol phase}}{\mathrm{counts in PBS phase}}$ . The radioactivity measurements were decay-corrected to the same time point for all samples.

### PET in Rats and Mice

All animal experiments were approved by the national Project Authorisation Board in Finland (license numbers ESAVI/3630/2023) and performed in compliance with the European Union (EU) Directive 2010/EU/63 on the protection of animals used for scientific purposes and Finnish national legislation (497/2013 Act). Healthy male Sprague–Dawley rats (*n* = 4; body weight 232.6 ± 14.4 g; age 7.1 ± 0.0 weeks) were obtained from the Central Animal Laboratory, University of Turku, Turku, Finland. PET/CT imaging was performed using an Inveon Multimodality scanner (Siemens Medical Solutions, Knoxville, TN, U.S.A.) following a previously published protocol.^19^ Rats were anesthetized with isoflurane (4–5% for induction, 1.5–2% for maintenance) and a tail vein cannula was inserted. High-resolution CT scans were acquired first for anatomical reference and attenuation correction, followed by dynamic 60 min PET acquisition after intravenous injection of [^18^F]­FNA–folate (10.27 ± 0.15 MBq, 400.0 ± 115.5 μL). PET data were acquired in a list-mode and reconstructed using the ordered subsets expectation maximization 3-dimensional (OSEM-3D) algorithm into 6 × 10 s, 4 × 60 s, 5 × 300 s, and 3 × 600 s time frames.

PET/CT imaging was performed using Molecubes small-animal PET and CT systems (Molecubes NV, Gent, Belgium) in mice (C57BL/6J, males; *n* = 8). [^18^F]­FNA–folate was injected via a tail vein cannula at a dose of 4.1 ± 0.4 MBq. Image analysis was performed using Carimas 2.10 software (Turku PET Centre, Turku, Finland; www.turkupetcentre.fi/carimas/). For anatomical reference, regions of interest (ROIs) were manually defined using CT. TACs were extracted from the 60 min PET data and expressed as SUVs.

Tissues were collected immediately after PET/CT imaging for *ex vivo* biodistribution measurements in four of the mice. Mice were euthanized by cardiac puncture under deep anesthesia followed by cervical dislocation. Blood was drawn from the left ventricle, and urine was collected simultaneously. In addition to blood and urine, the following tissues were collected: brain, muscle, blood, heart, lung, liver, spleen, pancreas, kidney, empty stomach, empty small intestine, empty large intestine, lymph nodes, skull bone, and femur bone with marrow. Tissues were weighed, and radioactivity was measured using a Wizard gamma counter (Wallac Oy, Turku, Finland). Tissue radioactivity concentration was expressed as %ID/g, with the injected dose corrected for residual radioactivity in the cannula and tail.

### Radioactivity Binding in Blood Components and Stability *In Vivo*

At the end of PET imaging with [^18^F]­FNA–folate, four of the mice were euthanized, and blood samples were collected from the left ventricle as described above. Blood was collected in heparinized tubes containing a gel layer to facilitate blood cell separation from plasma. Blood cells were isolated by centrifugation at 2100*g* for 5 min. Plasma was separated, and plasma proteins were precipitated by adding an equal volume of acetonitrile. Plasma proteins were pelleted by centrifugation at 14000*g* for 2 min, and the supernatants were transferred to separate Eppendorf tubes. The radioactivity in the blood cell fraction, plasma protein fraction, and plasma supernatant was quantified using a gamma counter and decay-corrected. To evaluate *in vivo* stability of [^18^F]­FNA–folate, plasma supernatant aliquots were analyzed by HPLC (LaChrom Instruments, Hitachi) equipped with a Radiomatic 150TR flow-through radioactivity detector (Packard). A reversed-phase C18 column (Jupiter Proteo, 250 × 10 mm, 5 μm, 90 Å; Phenomenex) was used at a flow rate of 5 mL/min. Solvent A was 0.1% TFA in water, and solvent B was 0.1% TFA in acetonitrile. Gradient elution was performed from 25 to 45% B over 1–16 min. The identity of the intact [^18^F]­FNA–folate was confirmed by comparison with the tracer standard analyzed under the same conditions.

### *In Vitro* Tissue Binding and Blocking with [^18^F]­FNA–Folate

*In vitro* binding and blocking studies were performed using cryosections (8 μm) from rats with myocardial infarction (license number ESAVI/43134/2019).^21^ Tissue sections were thawed at 4 °C for 15 min, warmed up to RT for 15 min, and preincubated in PBS at RT for 15 min before incubation with the radiotracer. Sections were then incubated in 100 mL PBS containing 1.9 MBq of [^18^F]­FNA–folate for 45 min. After incubation, the slides were washed twice with cold (4 °C) PBS (2 min each), rinsed once with cold water, dried with a gentle air stream, and exposed to phosphor imaging plates for 20 h. Autoradiographic signals were visualized using a Fujifilm BAS5000 phosphor imaging system. For blocking studies, adjacent tissue sections were incubated under identical experimental conditions, with the tracer solution supplemented with folate glucosamine (1 μM) as a competitive inhibitor. After autoradiography, the same slides were H&E stained to provide histological reference, while adjacent sections were used for CD68 immunohistochemistry.^21^

### Statistical Analysis

Where applicable, data are presented as mean ± standard deviation. Statistical differences were evaluated using the unpaired Student’s *t* test (Excel, Microsoft, U.S.A.). *p* values of <0.05 were considered statistically significant.

## Supplementary Material



## Notes

Animal study design (Figure S1) and *in vivo* biodistribution (Table S1) (PDF)

## Abbreviations Used

%ID/g: percentage of injected radioactivity dose per gram of tissue
[^18^F]­FNA–folate: fluorine-18-labeled 6-fluoronicotinic-acid-conjugated folate
^18^F: fluorine-18
CH_3_CN: acetonitrile
CT: computed tomography
DMSO: dimethyl sulfoxide
ESI–MS: electrospray ionization mass spectrometry
EtOH: ethanol
FNA: 6-fluoronicotinic acid
GMP: good manufacturing practice
H&E: hematoxylin and eosin
HLB: hydrophilic–lipophilic balance
HPLC: high-performance liquid chromatography
OSEM-3D: ordered subsets expectation maximization three-dimensional
PBS: phosphate-buffered saline
PET: positron emission tomography
ROI: region of interest
RT: room temperature
SFB: *N*-succinimidyl-4-fluorobenzoate
SUV: standardized uptake value
SUV_mean_: mean standardized uptake value
TAC: time–activity curve
TFA: trifluoroacetic acid

## References

[1] Xu X., Jané P., Taelman V., Jané E., Dumont R. A., Garama Y., Kim F., del Val
Gómez M., Gariani K., Walter M. A. (2024). The theranostic
genome. Nat. Commun..

[2] Wagner L., Kenzhebayeva B., Dhaini B., Boukhlef S., Moussaron A., Mordon S., Frochot C., Collet C., Acherar S. (2022). Folate-based
radiotracers for nuclear imaging and radionuclide therapy. Coord. Chem. Rev..

[3] Moore K. N., Angelergues A., Konecny G. E., García Y., Banerjee S., Lorusso D., Lee J.-Y. (2023). Mirvetuximab
Soravtansine in FRα-positive, platinum-resistant ovarian cancer. N. Engl. J. Med..

[4] Gnesin S., Müller J., Burger I. A., Meisel A., Siano M., Früh M., Choschzick M., Müller C., Schibli R., Ametamey S. M., Kaufmann P. A., Treyer V., Prior J. O., Schaefer N. (2020). Radiation dosimetry of ^18^F-AzaFol: a first in-human use of a folate receptor PET tracer. EJNMMI Res..

[5] Guzik P., Fang H.-Y., Deberle L. M., Benešová M., Cohrs S., Boss S. D., Ametamey S. M., Schibli R., Müller C. (2021). Identification
of a PET radiotracer for imaging of
the folate receptor-α: a potential tool to select patients for
targeted tumor therapy. J. Nucl. Med..

[6] Silvola J. M. U., Li X.-G., Virta J., Marjamäki P., Liljenbäck H., Hytönen J. P., Tarkia M., Saunavaara V., Hurme S., Palani S., Hakovirta H., Ylä-Herttuala S., Saukko P., Chen Q., Low P. S., Knuuti J., Saraste A., Roivainen A. (2018). Aluminum fluoride-18
labeled folate enables in vivo detection of atherosclerotic plaque
inflammation by positron emission tomography. Sci. Rep..

[7] Jahandideh A., Uotila S., Ståhle M., Virta J., Li X.-G., Kytö V., Marjamäki P., Liljenbäck H., Taimen P., Oikonen V., Lehtonen J., Mäyränpää M. I., Chen Q., Low P. S., Knuuti J., Roivainen A., Saraste A. (2020). Folate receptor β-targeted PET imaging of macrophages
in autoimmune myocarditis. J. Nucl. Med..

[8] Moisio O., Palani S., Virta J., Elo P., Liljenbäck H., Tolvanen T., Käkelä M., Miner M. G., Herre E. A., Marjamäki P., Örd T., Heinäniemi M., Kaikkonen M. U., Zhang F., Srinivasarao M., Knuuti J., Low P. S., Saraste A., Li X.-G., Roivainen A. (2020). Radiosynthesis
and preclinical evaluation of [^68^Ga]­Ga-NOTA-folate for
PET imaging of folate receptor β-positive
macrophages. Sci. Rep..

[9] Olberg D. E., Arukwe J. M., Grace D., Hjelstuen O. K., Solbakken M., Kindberg G. M., Cuthbertson A. (2010). One step radiosynthesis
of 6-[^18^F]­fluoronicotinic acid 2,3,5,6-tetrafluorophenyl
ester ([^18^F]­F-Py-TFP): a new prosthetic group for efficient
labeling of biomolecules with fluorine-18. J.
Med. Chem..

[10] Dillemuth P., Lövdahl P., Karskela T., Ayo A., Ponkamo J., Liljenbäck H., Paunonen S., Kunnas J., Rajander J., Tynninen O., Rosenholm J. M., Roivainen A., Laakkonen P., Airaksinen A. J., Li X.-G. (2024). Switching the chemoselectivity
in the preparation of [^18^F]­FNA-*N*-CooP,
a free thiol-containing peptide for PET imaging of fatty acid binding
protein 3. Mol. Pharmaceutics.

[11] Dillemuth P., Karskela T., Ayo A., Ponkamo J., Kunnas J., Rajander J., Tynninen O., Roivainen A., Laakkonen P., Airaksinen A. J., Li X.-G. (2024). Radiosynthesis,
structural identification and in vitro tissue binding study of [^18^F]­FNA-*S*-ACooP, a novel radiopeptide for
targeted PET imaging of fatty acid binding protein 3. EJNMMI Radiopharm. Chem..

[12] Keam S. J. (2021). Piflufolastat
F 18: diagnostic first approval. Mol. Diagn.
Ther..

[13] Giesel F. L., Hadaschik B., Cardinale J., Radtke J., Vinsensia M., Lehnert W., Kesch C., Tolstov Y., Singer S., Grabe N., Duensing S., Schäfer M., Neels O. C., Mier W., Haberkorn U., Kopka K., Kratochwil C. (2017). F-18 labelled PSMA-1007: biodistribution,
radiation dosimetry and histopathological validation of tumor lesions
in prostate cancer patients. Eur. J. Nucl. Med.
Mol. Imaging..

[14] Dillemuth P., Ayo A., Zhuang X., Lövdahl P., Liljenbäck H., Kärnä S., Auchynnikava T., Kunnas J., Ponkamo J., Miner M. W. G., Rajander J., Rosenholm J. M., Roivainen A., Airaksinen A. J., Laakkonen P., Li X.-G. (2025). Rapid cleavage of 6-[^18^F]­fluoronicotinic acid prosthetic
group governs BT12 glioblastoma xenograft uptake: implications for
radiolabeling design of biomolecules. EJNMMI
Radiopharm. Chem..

[15] Li X.-G., Helariutta K., Roivainen A., Jalkanen S., Knuuti J., Airaksinen A. J. (2014). Using 5-deoxy-5-[18F]­fluororibose
to glycosylate peptides
for positron emission tomography. Nat. Protoc..

[16] Basuli F., Zhang X., Jagoda E. M., Choyke P. L., Swenson R. E. (2016). Facile
room temperature synthesis of fluorine-18 labeled fluoronicotinic
acid-2,3,5,6-tetrafluorophenyl ester without azeotropic drying of
fluorine-18. Nucl. Med. Biol..

[17] Haskali M. B., Farnsworth A. L., Roselt P. D., Hutton C. A. (2020). 4-Nitrophenyl activated
esters are superior synthons for indirect radiofluorination of biomolecules. RSC. Med. Chem..

[18] Paladugula N., Fazili Z., Sternberg M. R., Gabey G., Pfeiffer C. M. (2019). Serum folate
forms are stable during repeated analysis in the presence of ascorbic
acid and during frozen sample storage. J. Appl.
Lab Med..

[19] Dillemuth P., Ayo A., Airenne T. T., Lövdahl P., Bakay E., Zhuang X., Liljenbäck H., Paunonen S. T., Kunnas J., Filppu P., Rajander J., Johnson M. S., Roivainen A., Salminen T. A., Rosenholm J. M., Laakkonen P., Li X.-G. (2025). Utilizing monocarboxylate transporter 1-mediated blood-brain barrier
penetration for glioblastoma positron emission tomography imaging
with 6-[^18^F]­fluoronicotinic acid. Mol. Pharmaceutics.

[20] Bauer M., Karch R., Wulkersdorfer B., Philippe C., Nics L., Klebermass E.-M., Weber M., Poschner S., Haslacher H., Jäger W., Tournier N., Wadsak W., Hacker M., Zeitlinger M., Langer O. (2019). A proof-of-concept study to inhibit
ABCG2- and ABCB1-mediated efflux transport at the human blood-brain
barrier. J. Nucl. Med..

[21] Andriana P., Palani S., Liljenbäck H., Iqbal I., Oikonen V., Virta J., Makrypidi K., Rajander J., Herre E. A., Suni A., Jalkanen S., Knuuti J., Martinez-Pomares L., Pirmettis I., Li X.-G., Saraste A., Roivainen A. (2025). Macrophage
mannose receptor CD206-targeted PET imaging in experimental acute
myocardial infarction. EJNMMI Res..

[22] Verweij N. J. F., Yaqub M., Bruijnen S. T. G., Pieplenbosch S., ter Wee M. M., Jansen G., Chen Q., Low P. S., Windhorst A. D., Lammertsma A. A., Hoekstra O. S., Voskuyl A. E., van der Laken C. J. (2020). First in
man study of [^18^F]­fluoro-PEG-folate
PET: a novel macrophage imaging technique to visualize rheumatoid
arthritis. Sci. Rep.

