# Supplementary material for: [18F]Fluoronicotinic-Acid-Conjugated Folate as a Novel Candidate Positron Emission Tomography Tracer for Inflammation
Source: ACS Omega. 2025 Dec 31;11(1):1898–907. doi: 10.1021/acsomega.5c10157 (PMC12809762; doi:10.1021/acsomega.5c10157)
Supplement: Supplementary file 1 [file ao5c10157_si_001.pdf]

Supporting Information

[^{18}F]Fluoronicotinic acid-conjugated folate as a novel candidate PET tracer for inflammation

Xiaoqing Zhuang,[a,b,c, #] *Jonne Kunnas,*[a,d, #] *David Ekwe,*[a,b,d] *Emel Bakay,*[a,d] *Pyry Dillemuth,*[a,b,c] *Heidi Liljenbäck,*[a] *Imran Iqbal,*[a] *Johan Rajander,*[a,e] *Philip S. Low,*[f] *Juhani Knuuti,*[a,b,g,h] *Jessica M. Rosenholm,*[d] *Antti Saraste,*[a,b,g,h] *Anne Roivainen,*[a,b,g,i] *Xiang-Guo Li*[a,b,c,g*]

[a]Turku PET Centre, University of Turku, Finland

[b]Turku PET Centre, Turku University Hospital, Finland

[c]Department of Chemistry, University of Turku, Finland

[d]Pharmaceutical Sciences Laboratory, Department of Natural and Health Sciences, Faculty of Science and Engineering, Åbo Akademi University, Turku, Finland

[e]Accelerator Laboratory, Åbo Akademi University, Turku, Finland

[f]Department of Chemistry, Purdue University, West Lafayatte, IN, United States

[g]InFLAMES Research Flagship Center, University of Turku, Finland

[h]Heart Center, Turku University Hospital, Turku, Finland

[i]Turku Center for Disease Modeling, University of Turku, Turku, Finland

[#] Authors with equal contribution.

***Corresponding Author:** Associate Professor Xiang-Guo Li, PhD, Turku PET Centre, Kiinamyllynkatu 4-8, FI-20520 Turku, Finland; Phone: +358504485069; E-mail: xiali@utu.fi

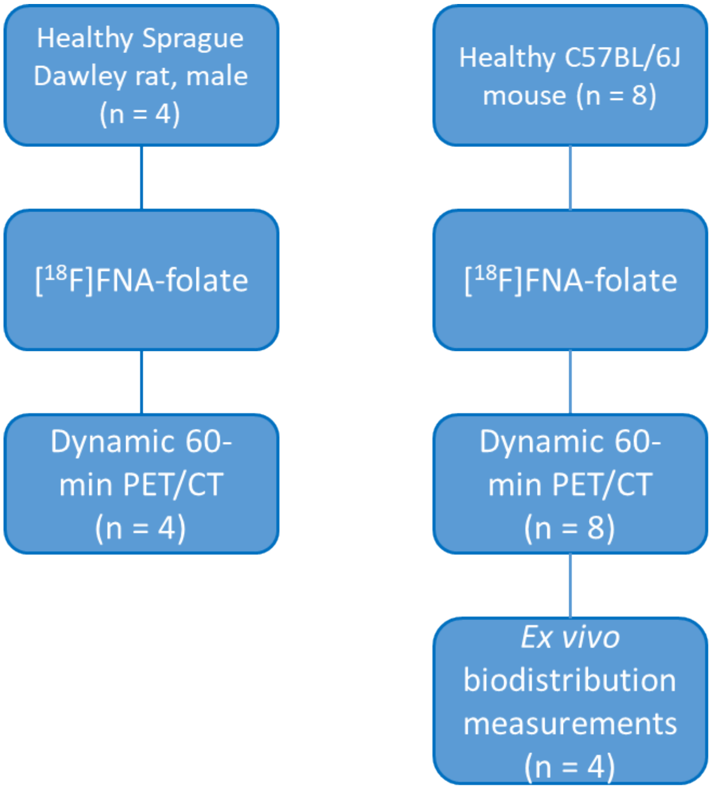

Figure S1. Animal study design.

Table S1. *In vivo* biodistribution of [^{18}F]FNA-folate in healthy C57BL/6J male mice based on 60-min dynamic PET imaging.

Radioactivity concentration (%ID/mL)*							
**Time**		**Heart**	**Muscle**	**Brain**	**Kidneys**	**Lungs**	**Liver**
**Time (seconds)**	0 - 10	2.23 ± 4.06	0.04 ± 0.07	0.12 ± 0.27	0.34 ± 0.63	0.73 ± 1.24	0.32 ± 0.47
	10 - 20	10.77 ± 10.43	0.41 ± 0.50	0.83 ± 0.86	3.51 ± 3.58	4.25 ± 3.58	2.08 ± 1.98
	20 - 30	11.60 ± 7.97	0.73 ± 0.49	0.96 ± 0.66	6.73 ± 4.58	5.01 ± 2.87	4.77 ± 4.70
	30 - 40	9.52 ± 5.75	0.78 ± 0.49	0.93 ± 0.51	8.31 ± 4.72	4.57 ± 2.62	8.10 ± 6.88
	40 - 50	11.74 ± 7.04	1.09 ± 0.44	1.01 ± 0.56	10.38 ± 4.68	5.54 ± 2.76	11.38 ± 7.45
	50 - 60	8.54 ± 5.32	1.23 ± 0.57	0.90 ± 0.50	11.98 ± 5.55	4.59 ± 2.26	15.32 ± 8.39
**Time (minutes)**	1 - 2	5.48 ± 2.77	1.05 ± 0.33	0.63 ± 0.24	13.52 ± 5.25	3.49 ± 0.71	22.79 ± 10.39
	2 -3	3.33 ± 1.98	0.91 ± 0.27	0.44 ± 0.20	14.53 ± 3.96	2.87 ± 0.85	26.64 ± 6.60
	3 - 4	2.45 ± 0.73	0.76 ± 0.31	0.34 ± 0.10	13.99 ± 4.08	2.50 ± 0.76	24.00 ± 4.91
	4 - 5	2.17 ± 0.81	0.62 ± 0.16	0.31 ± 0.09	14.13 ± 4.06	2.30 ± 1.04	19.75 ± 4.79
	5 - 10	1.99 ± 0.85	0.47 ± 0.14	0.27 ± 0.09	14.47 ± 3.83	2.07 ± 0.86	13.96 ± 3.70
	10 - 15	1.39 ± 0.52	0.30 ± 0.06	0.21 ± 0.06	14.77 ± 3.55	1.36 ± 0.38	7.43 ± 0.84
	15 - 20	1.13 ± 0.40	0.21 ± 0.06	0.19 ± 0.05	15.47 ± 3.72	1.14 ± 0.28	6.45 ± 1.46
	20 - 25	1.08 ± 0.39	0.17 ± 0.05	0.18 ± 0.05	15.98 ± 3.82	1.03 ± 0.23	6.55 ± 2.10
	25 - 30	0.97 ± 0.41	0.12 ± 0.04	0.16 ± 0.05	16.64 ± 3.74	0.93 ± 0.25	6.52 ± 2.61
	30 - 40	0.95 ± 0.45	0.13 ± 0.03	0.15 ± 0.06	17.54 ± 4.29	0.84 ± 0.29	6.72 ± 3.10
	40 - 50	0.92 ± 0.45	0.10 ± 0.03	0.14 ± 0.05	16.89 ± 4.65	0.75 ± 0.24	7.55 ± 3.50
	50 - 60	0.94 ± 0.58	0.08 ± 0.03	0.12 ± 0.05	16.52 ± 5.00	0.70 ± 0.20	6.92 ± 4.02

*Results are presented as mean ± standard deviation (n = 8).
